# Vacuum Low-Temperature Microwave-Assisted Pyrolysis of Technical Lignins

**DOI:** 10.3390/polym14163383

**Published:** 2022-08-18

**Authors:** Johannes Karthäuser, Vladimirs Biziks, Holm Frauendorf, Carsten Mai, Holger Militz

**Affiliations:** 1 Department of Wood Biology and Wood Products, Georg-August University of Goettingen, Buesgenweg 4, 37077 Goettingen, Germany; 2 Surfactor Germany GmbH, Braunschweiger Str. 23 b, 38170 Schoeppenstedt, Germany; 3 Institute of Organic and Biomolecular Chemistry, Georg-August University of Goettingen, Tammannstraße 2, 37077 Goettingen, Germany

**Keywords:** lignin, pyrolysis, microwave pyrolysis, bio-oil

## Abstract

Cleavage by microwave-assisted pyrolysis is a way to obtain higher-value organic chemicals from technical lignins. In this report, pine kraft lignin (PKL), spruce and beech organosolv lignin (SOSL and BOSL), and calcium lignosulfonates from spruce wood (LS) were pyrolyzed at temperatures between 30 and 280 °C using vacuum low-temperature, microwave-assisted pyrolysis. The mass balance, energy consumption, condensation rate, and pressure changes of the products during the pyrolysis process were recorded. Phenolic condensates obtained at different temperatures during pyrolysis were collected, and their chemical composition was determined by GC-MS and GC-FID. The origin of the technical lignin had a significant influence on the pyrolysis products. Phenolic condensates were obtained in yields of approximately 15% (PKL and SOSL) as well as in lower yields of 4.5% (BOSL) or even 1.7% (LS). The main production of the phenolic condensates for the PKL and SOSL occurred at temperatures of approximately 140 and 180 °C, respectively. The main components of the phenolic fraction of the three softwood lignins were guaiacol, 4-methylguaiacol, 4-ethylguaiacol, and other guaiacol derivatives; however, the quantity varied significantly depending on the lignin source. Due to the low cleavage temperature vacuum, low-temperature, microwave-assisted pyrolysis could be an interesting approach to lignin conversion.

## 1. Introduction

Lignin is the most abundant aromatic polymer and the second most abundant bio-mass on Earth [1,2]. During pulp production and other processes, lignin is obtained as a side-product, referred to as technical lignin. Well-known examples of technical lignins are kraft lignin, organosolv lignin, and lignosulfonates, with kraft pulping currently being the dominant process for pulp production. During kraft pulping, the lignocellulosic biomass is exposed to high temperatures, pressure, and basic conditions due to the presence of sodium hydroxide and sodium sulfide reactants. Organosolv lignin is cleaved in organic solvents under harsh conditions [3]. Lignosulfonates are obtained similar to kraft lignin but with the sulfite reactants mostly in an acidic medium [4,5]. In all of these cases, the process leads to cleavage and recondensation of the natural lignin. A high portion of the *β*-O-4 bonds, which are dominant in natural lignin, are cleaved, and numerous different new C-C bonds emerge [3,6]. Due to the fact of this, the resulting structure of technical lignins is recalcitrant, complex, and heterogeneous [3,7]. Hence, 95% of technical lignins are burned to supply energy and regain inorganic pulping chemicals or are even discarded [8].

Searching for higher-value applications of technical lignin, research interest in cleaving lignin has been high, with an advantage being the higher reactivity of the resulting monomers [5,9]. For this, several methods have been proposed. A promising approach is pyrolysis, during which lignin is thermally treated in an oxygen-free atmosphere [7]. Herein, methods were divided into conventional heating pyrolysis and microwave-assisted pyrolysis. Microwave-assisted pyrolysis is a more recent approach that aims to improve the energy efficiency and heat the sample more uniformly [10]. Most research on microwave-assisted pyrolysis of lignin describes the pyrolysis processes heating up to temperatures of 500 °C or higher [10,11,12,13,14,15,16]. Currently, there are few publications regarding microwave-assisted lignin pyrolysis at lower temperatures. Bu et al. (2014) [17] reported the microwave-assisted pyrolysis of alkali lignin at temperatures of 350–550 °C under a nitrogen atmosphere using activated carbon as a catalyst. With most of the settings, bio-oil yields of 18–27% were obtained; however, the maximum bio-oil yield was 41.5%. The bio-oils had a water content of approximately 20–30% and contained phenols and guaiacols as the main components [17]. Bartoli et al. (2020) [18] pyrolyzed kraft lignin at 350–450 °C using microwave-absorbing materials as catalysts and varying the pressure applied during pyrolysis. At 450 °C, bio-oil yields of up to 44% with a moisture content of 27% and an aromatic fraction of 53% were obtained [18]. Nde et al. (2021) [19] described microwave-assisted pyrolysis of kraft lignin at different temperatures between 300 and 700 °C under a nitrogen atmosphere. The highest bio-oil yields were obtained at 500 °C with 11%, while at 400 °C the yield was approximately 10% and at 300 °C approximately 5%. The main components of the bio-oil were guaiacols and phenols [19].

In this publication, pyrolysis products of pine kraft lignin (PKL), spruce organosolv lignin (SOSL), beech organosolv lignin (BOSL), and softwood calcium lignosulfonates (LS) pyrolyzed with vacuum low-temperature, microwave-assisted pyrolysis are described. The pyrolysis was performed without catalysts at temperatures up to 280 °C. This publication focused on the characterization of the organic condensate fraction.

## 2. Materials and Methods

### 2.1. Materials

The technical lignins investigated were PKL with the trademark “Lineo^TM^” from Stora Enso (Stora Enso Oyj, Helsinki, Finland), BOSL and SOSL from Fraunhofer CBP (Fraunhofer Center for Chemical-Biotechnological Processes CBP, Leuna, Germany), and LS from Borregaard (Borregaard AS, Sarpsborg, Norway).

### 2.2. Pyrolysis of Lignin

**Figure 1:**
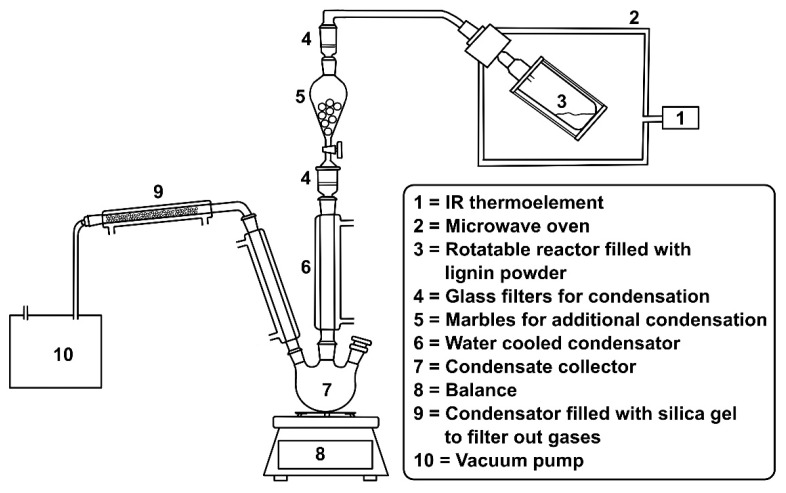
Schematic representation of the pyrolysis setup.

The lignin was obtained as a powder. In the case of SOSL and BOSL, the powder was partly sticking together, resulting in larger particles up to 1 cm in diameter. The lignin powder was dried at 50 °C for 24 h before pyrolysis. This was done because higher moisture levels lead to more lignin being carried into the condensation system by water. The low temperature was selected to decrease the chemical alterations in the lignin sample. A schematic representation of the pyrolysis setup can be found in Figure 1. The pyrolysis was carried out using a “Milestone flexiWAVE” reactor (Microwave Systems (MWS), Leutkirch, Germany). For each measurement, 150 g of lignin sample were placed into a 2.7 L quartz glass reactor. Applying a vacuum, the lignin was heated from 30 to 280 °C while slowly rotating. The material was heated to 110 °C at a heating rate of 3.5 °C/min and then to 280 °C at 5 °C/min. The temperature was kept at 280 °C for two minutes after heating. Visually and considering the condensate production rate, it was made sure that after the two minutes, no more significant production of products occurred. The low heating rates of 3.5 °C/min up to 110 °C were selected to ensure that the equipment would not be damaged by too fast heating and to decrease the amount of powder being carried into the condensation system. At temperatures above 110 °C, where cleavage reactions were occurring, the higher heating rate was applied. Afterwards, the reactor was cooled down to 40 °C. The temperature was measured with an infrared thermoelement. At the start of the pyrolysis process, the pressure was decreased to approximately 50 mbar. While heating, the pressure changed depending on the reactions taking place in the reactor. The temperature, vacuum pressure and energy consumption during the pyrolysis process were recorded by the control panel. The condensate production was monitored by placing the condensation system on a balance and manually collecting the mass every 10 min. All volatile compounds were evacuated from the reactor with the help of a vacuum pump. Quartz glass filters and silica gel were used to increase the condensation rate. After pyrolysis, the mass of the remaining char and the mass of the condensate was measured. The mass of the noncondensable gases was determined by subtracting the mass of the char and condensate from the mass of the starting material. All lignins were pyrolyzed in at least two replicates, except for the SOSL, which was pyrolyzed only once due to the fact of its limited availability.

After pyrolyzing, the liquid product was separated into two phases, referred to as an aqueous phase and a phenolic phase/bio-oil. The separation was achieved by liquid–liquid fractionation in a separation funnel using trichloromethane (TCM; >99.9%, Th. Geyer GmbH & Co. KG, Renningen, Germany). The TCM was removed from the phenolic phase with a rotary evaporator.

A heavy organic, tar-like fraction was extracted from the pyrolysis char by acetone extraction (>99%, Th. Geyer GmbH & Co. KG, Renningen, Germany). For this, the char was mechanically broken into smaller pieces and submerged in acetone. The acetone extract was filtered from the char. It was then evaporated to obtain the dark, viscous substance. The process was repeated until the acetone extract had a more transparent, less dark appearance. Additionally, the mass of the obtained extract was considered a factor for determining when the extraction was complete. The viscous substance is referred to as “tar”, while the extracted char is referred to as “coke”.

### 2.3. Temperature Series Condensates of Pine Kraft and Spruce Organosolv Lignin

To learn more about the pyrolysis process, samples of condensates were collected at 118, 127, 136, 180, and 280 °C. These temperatures were selected based on the condensate production rate measured during pyrolysis. Condensates were collected at temperatures at which high condensate production took place. Condensates were obtained in a glass filter filled with cotton, which was exchanged at the desired temperature. The mass of the collected condensates was determined, and the condensates were eluted from the cotton with acetone (>99%, Th. Geyer GmbH & Co. KG, Renningen, Germany). After evaporating the acetone, the liquids were analyzed by GC-MS and GC-FID.

### 2.4. Analytical Methods

#### 2.4.1. Thermogravimetric Analysis (TGA)

TGA was performed on a TG 209 ***F1*** *Iris*^TM^ (NETZSCH Gerätebau GmbH, Selb, Germany). Lignin was dried overnight at 60 °C. Approximately 10 mg of lignin was weighed into an Al_2_O_3_ crucible. The crucible was heated from 50 °C up to 700 °C, with a heating rate of 20 °C/min under a nitrogen gas flow of 20 mL/min. The percentage mass loss was determined while heating.

#### 2.4.2. Fourier-Transform Infrared Spectroscopy (FTIR)

FTIR was conducted on an Alpha FTIR Spectrophotometer (Bruker Optik GmbH, Bremen, Germany) in the attenuated total reflection (ATR) mode. The measurements were performed at room temperature between wavenumbers of 4000 and 400 cm^−1^ with a resolution of 4 cm^−1^. The samples were measured with 64 scans. Baseline corrections of the spectra were automatically performed by the baseline correction tool integrated into the software. The peaks were normalized by dividing through the highest measured signal intensity. The peak maximum was determined manually using OriginPro 8.5 G (OriginLab, Northampton, MA, USA).

#### 2.4.3. Gas Chromatography-Mass Spectrometry (GC-MS)

The qualitative composition of the bio-oils was determined by GC-MS using a DSQ mass spectrometer equipped with a Trace GC gas chromatograph provided by Thermo Electron Corporation (Waltham, MA, USA) using a VF-5 ms capillary gas chromatography column (Agilent J&W; length: 30 m, ID: 0.25 mm, and d_f_: 0.25 µm). The solvent solution was prepared by adding 20 mg of iodobenzene (>98%, Acros Organics Bvba, Geel, Belgium) as an internal standard to 20 mL of acetone (≥99.8%, Merck KGaA, Darmstadt, Germany). Iodobenzene was chosen as an internal standard, because it was not present in the lignin samples, contains an aromatic ring system, and produces only a few fragment ions. Furthermore, there was no coelution with one of the major bio-oil constituents. A quantity of 10 µL of bio-oil was diluted with 990 µL of this solution. After introducing 0.5 µL of the sample with a programmed temperature vaporization (PTV) injector set constantly to a temperature of 250 °C and a split ratio of 1:10, the temperature in the GC column was increased from 50 to 250 °C, applying a heating rate of 15 °C/min. The NIST and Wiley mass spectra databases were used to support the structural identification. The match factors for the proposed assignments were at least 800 for the similarity index (SI) as well as the reversed search index (RSI).

#### 2.4.4. Gas Chromatography-Flame Ionization Detector (GC-FID)

To determine the quantity of the different components in the bio-oil, a 7890A GC System with a 7683B Series Injector (both Agilent Technologies, Santa Clara, CA, USA) was used. Gas chromatographic separation was performed applying a DB-5MS GC column (30 m × 0.25 mm, d_f_: 0.25 µm, Agilent Technologies, Santa Clara, CA, USA). The samples were prepared in a similar way as described for the GC-MS measurements. One microliter of the samples was injected into a split/splitless injector at 250 °C with a split ratio of 1:20, and the GC column was heated from 50 to 250 °C with a heating rate of 15 °C/min. The peak area used for quantification of the substances was determined automatically in the tangent skim mode. For quantification, standard solutions containing the five main constituents of the bio-oil, selected by peak area, were prepared. For this, concentrations of 0.5, 0.25, and 0.1 mg/mL of guaiacol (98%, Carl Roth GmbH + Co. KG, Karlsruhe, Germany), 4-methyl guaiacol (99%, J&K Scientific Ltd., Beijing, China), 4-ethyl guaiacol (98.1%, HPC Standards GmbH, Cunnersdorf, Germany), 4-propyl guaiacol (99%, Sigma-Aldrich, Co., St. Louis, MO, USA), and 4-(1-propenyl) guaiacol (95%, Fluorochem Ltd., Hadfield, UK) diluted in acetone were prepared. Adding 1 mg/mL of iodobenzene to each of the standard solutions, the ratio between the peak area of each of the other compounds to iodobenzene was calculated. A calibration line was determined, and the quantity of the compounds in the samples were calculated with the internal standard iodobenzene. The mass percent of the compounds in the bio-oil was determined using the density of the bio-oils. A volume of 200 µL of bio-oil was collected using a micropipette. The density was calculated based on the mass difference. This was repeated three times, and the average value was used.

## 3. Results and Discussion

### 3.1. Characterization of the Original Lignins Using FTIR

**Figure 2:**
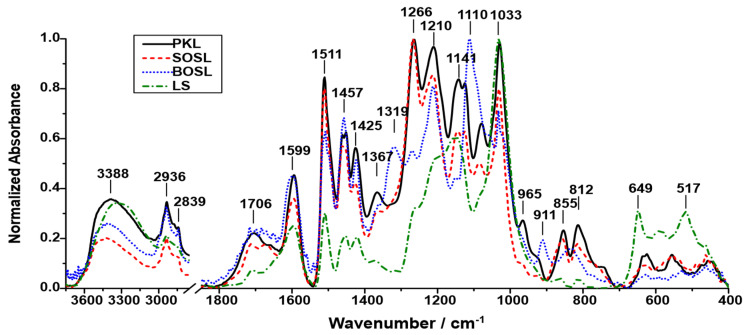
Normalized FTIR spectra of the four untreated technical lignins: LS (green; dashes–dots); BOSL (blue; dots); SOSL (red; dash); PKL (black; solid line).

To obtain information regarding the chemical composition of the four lignins, they were analyzed using FTIR (Figure 2). The signals detected for the lignins occurred at similar wavenumbers; however, the relative intensity varied depending on the lignin type.

The proposed assignments of the signals are listed in Table 1.

The FTIR results provide insight into the chemical composition of the lignin; however, they must be examined with care. Due to the high complexity of the compound and the resulting high variety of functional groups, the signals overlapped, and a true baseline could not be determined. Thus, the real intensity of the signals could not be determined, and the results should only be considered for observing tendencies and not precise quantities. Despite this, some conclusions may be obtained from the FTIR spectra.

While the positions of the signals were roughly similar among the different lignins, the intensity varied, indicating that the abundance of the different functional groups producing the signals depends on the lignin source. The LS was the lignin that differed the most from the other lignins. In the spectrum recorded for LS, the highest intensity signal was 1033 cm^−1^. It could originate from aromatic in-plane deformation of G-type lignin or C=O stretching in lignin. As the LS was extracted from softwood, it is likely that G-type building blocks were the most abundant ones, making this the most probable signal source. Other pronounced signals for the LS were at 1141, 1511, and 1599 cm^−1^, originating from C-H in-plane deformation or phenolic OH, aromatic skeletal vibrations (G- > S-type lignin), and aromatic skeletal vibrations (S- > G-type lignin), respectively. Additionally, the broad signal at 3388 cm^−1^ originating from O-H stretching had a high intensity. This can be explained by the hydrophilicity of LS, which led to more water molecules being enclosed by the lignin. The signal at 649 cm^−1^ represents the sulfonate groups, which are characteristic of LS. The signals originating from the other functional groups were less pronounced than for the other lignins.

The BOSL is the only hardwood lignin examined in this study. Hence, signals originating from functional groups of S-type lignin have higher intensities compared to the other softwood lignins, which can be seen, for example, by the most pronounced signal at 1110 cm^−1^, originating from the C-H in-plane deformation of S-type lignin. However, there were also signals indicating a high number of G-type lignin structures such as the signal at 1210 cm^−1^ (C-C and C-O stretching in G-type lignin) or at 1511 cm^−1^ (aromatic skeletal vibrations G- > S-type lignin).

Compared to the spectrum of BOSL, the spectrum of SOSL had less significant signals at 1110 and 1457 cm^−1^. Strong signals were observed at wavenumbers of 1266, 1210, 1511, and 1033 cm^−1^. The signals at 1210 and 1511 cm^−1^, as well as one of two potential assignments of the signal at 1033 cm^−1^, were more typical for G-type lignin, which was to be expected considering the softwood origin of the SOSL. The highest signal at 1266 cm^−1^ was produced by C=O stretching vibrations in the lignin structures.

In the spectrum of the PKL, most of the signals with high intensity were similar to those of the SOSL. The main differences were that for the PKL, almost all signals had a higher intensity relative to the highest peak than for the SOSL. Considering that the spectrum depicts the normalized absorbance, the reason for this is that the abundance of the different functional groups was more uniform than in the SOSL, resulting in not one clear maximum signal upon normalization but various more similar ones.

### 3.2. Characterization of the Original Lignins Using TGA

**Figure 3:**
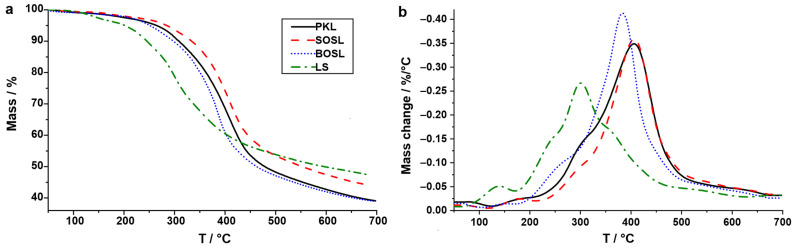
Mass (**a**) and mass change (**b**) as a function of the temperature of the four technical lignins: LS (green; dashes–dots); BOSL (blue; dot); SOSL (red; dash); PKL (black; solid line).

TGA was conducted on the technical lignins to analyze their thermal decomposition (Figure 3). The LS decomposed to a higher degree at earlier temperatures; however, they had a lower overall mass loss compared to the other lignins. At lower temperatures of up to approximately 200 °C, the mass loss of the LS amounted to approximately 3.7%. The total mass loss at 700 °C was 53.1%. Unlike for the LS, the mass loss of the other lignins occurred at similar temperatures and had a similar curved shape. However, the SOSL had a lower total mass loss than the PKL and the BOSL. The mass loss of up to 200 °C for the PKL, SOSL, and BOSL was approximately 2%. The total mass loss of SOSL was 56.4%, while for the BOSL and PKL, it was 61.1% and 60.9%, respectively.

The highest mass loss of the SOSL and PKL occurred at similar temperatures of approximately 405 °C. The BOSL had the highest decomposition rate at approximately 385 °C. As described above, the decomposition of the LS mainly occurred at lower temperatures, with the maximum occurring at approximately 290 °C.

As described above, the LS decomposed differently from the other lignins. First, there was a larger mass loss at lower temperatures. The reason for this might be that LS is soluble in water due to the hydrophilic functional groups; therefore, more water is still aggregated to these functionalities after drying. In addition, the cleavage of functional groups, such as SO_2_, ammonia, and water, take place at these lower temperatures for the LS [27]. Higher yields of these water-soluble products are also expected during microwave pyrolysis. The lower final mass loss of the LS could be due to the high calcium content. The data provide a first impression regarding the decomposition of the different technical lignins; however, the results of the decomposition by microwave-assisted pyrolysis were expected to be different, because the different heating and energy transfer mechanism cleaves bonds at potentially lower temperatures and yields different products than classical thermal heating [10]. In addition, the different atmospheres (namely, nitrogen flow, or vacuum) probably influence the cleaving process.

### 3.3. Observations on the Pyrolysis Products

**Figure 4:**
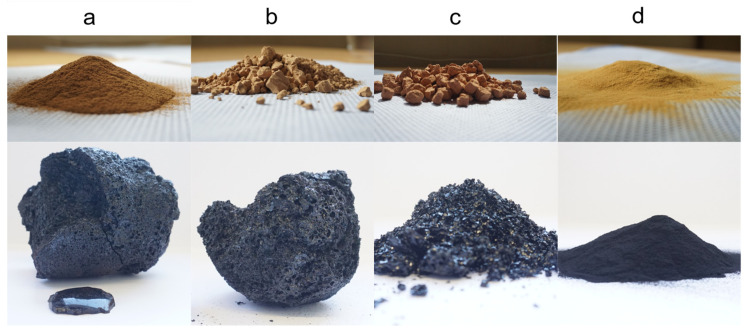
Original lignin and solid pyrolysis residue: (**a**) PKL; (**b**) SOSL; (**c**) BOSL; (**d**) LS.

Due to the applied vacuum and gaseous products acting as carriers, small quantities of lignin powder were transferred to the condensation system, which were separated during the liquid–liquid phase extraction step. The phenolic fractions of the different lignins had a dark brown color and a strong odor. The aqueous phases had a light brown color. The pyrolysis char had different appearances depending on the lignin type (Figure 4). The LS char was obtained as a dark powder; optically, compared to the original lignin, only the color changed (Figure 4d). The BOSL char was obtained as larger, dark, glassy particles (Figure 4c). The SOSL and PKL chars were obtained as foam-like, porous solid materials (Figure 4a,b). At the bottom of the PKL char, a glass-like layer formed.

The powder-like structure of the LS char after pyrolysis indicated that the lignin did not melt and that fewer new bonds were formed between the lignin molecules. This was opposite for the SOSL and PKL, where the molten lignin seemed to react, resulting in new bonds between the lignin molecules and, thus, a porous network. Additionally, the glassy layer in the PKL indicated that some of the molten material did not react in this char network and was not cleaved to molecules small enough to transfer into the condensation system. This observation of the glass-like layer also accounts for the BOSL. The lignin was probably molten during the pyrolysis; however, fewer crosslinking reactions took place. The reason for this could be the lower reactivity of the S-type lignin, which did not allow for the 5-O-4 or 5-5 condensation between the monomers.

### 3.4. Influence of the Lignin Origin on the Products

**Figure 5:**
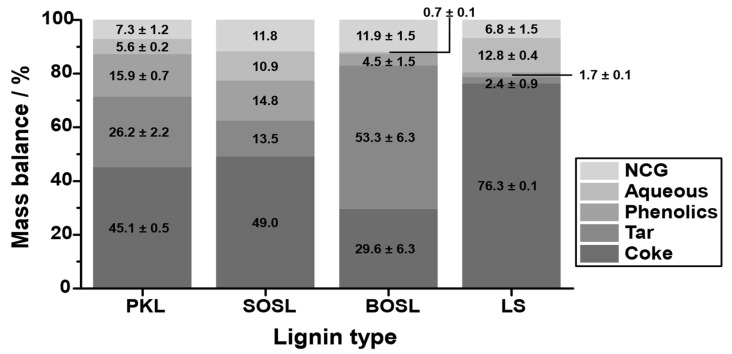
Mass balance including coke (very dark grey), tar (dark grey), phenolics (grey), aqueous fraction (light grey), and noncondensable gases (NCGs; very light grey) of the four technical lignins, PKL, SOSL, BOSL, and LS, after microwave-assisted pyrolysis.

The products were separated into an aqueous phase, a light organic (phenolic) phase, a noncondensable gas (NCG) phase, a heavy organic (tar-like) phase, and coke. The quantity of the different fractions varied significantly among the different lignins (Figure 5). While the coke yield of the PKL and the SOSL was similar at 45.1–49.0% and that of the BOSL was lower (29.6%), much more coke was obtained during the pyrolysis of the LS (76.3%). This higher coke yield was mainly compensated for by lower phenolics and tar production—together only approximately 4%. These yields were by far the lowest among the investigated technical lignins. Only 1.7% phenolics were obtained during the pyrolysis of LS. The phenolic fraction of the BOSL was slightly larger at 4.5%; however, it was still much lower than those of the PKL and SOSL, which were at 15.9% and 14.8%, respectively. The aqueous fraction of the LS was the largest together with the SOSL (12.8% and 10.9%), while the BOSL pyrolysis produced almost no aqueous products (0.7%).

The decomposition yields differed significantly from those obtained by TGA, especially considering the low temperature applied. This underlines the differences in the heating mechanism and their influence on the cleavage mechanism [10].

Comparing the three softwood lignins, an important factor on the mass balance could be the molecular mass. Kraft and organosolv lignins usually have a significantly lower molecular mass than lignosulfonates [4]. For smaller molecules, fewer bonds have to be cleaved to obtain phenolic chemicals that can be obtained as condensates. This could explain the high amount of coke for the LS as well as the higher phenolic yields for the BOSL, SOSL, and PKL. However, the phenolic yield of the BOSL was much lower than that of the PKL and SOSL. This indicates that the molecular mass was only one of many influences on the obtained products. Due to the hardwood origin of the BOSL, the structure of it was different to the SOSL, which was probably the reason for the different mass balances. In general, due to the lower reactivity of the S-type lignin, the natural hardwood lignins were more linear than the softwood lignins and contained fewer C-C bonds [6]. However, the characteristics of hardwood and softwood lignins after organosolv treatment depend on the treatment conditions and can vary significantly [28]. Thus, it is difficult to generally compare softwood and hardwood organosolv lignins. Considering the obtained results, the main difference between the organosolv lignins was that for the BOSL, a much larger tar fraction was obtained instead of the phenolics and aqueous phases.

The aqueous or water-soluble fraction was obtained in different yields depending on the lignin type. To determine the remaining organic molecules in the aqueous fraction, the fractions were analyzed using GC-FID. However, the detected quantity of carbon-containing molecules was negligible for all lignins. Comparing the aqueous yields with the mass loss up to 200 °C measured with TGA indicated that, especially for the LS and SOSL, the aqueous fraction was, to a large degree, not just bound water but originated from chemical cleavage. In the TGA, only approximately 2.0% or 3.7% (SOSL and LS, respectively) of mass loss by water at lower temperatures was detected, while the aqueous fraction in these cases comprised more than 10% of the mass balance. From the lignins that were used in this research, only the LS was water soluble. It has more hydrophilic groups, and it could be that despite the drying step before pyrolysis, bound water was still present and later on found in the aqueous fraction. Additional mass in the aqueous phase probably originated from the cleavage of hydroxy groups or other water-soluble functional groups. A reason for the higher yield in the aqueous phase for LS could be that fewer monomers were cleaved and obtained as condensates. Thus, more functional groups were still present in the reactor at higher temperatures, allowing further cleavage of small molecules, which are soluble in water. Varying yields among the remaining lignins were probably due to the chemical structure of the lignins.

There are several factors that can influence the NCG yield. Because the gases were not collected in the experimental setup used herein, direct analysis was not possible. Due to the vacuum pump system, portions of water were sucked into the pumping system and afterwards condensed only at the exit. This water was also considered as part of the NCG fraction. Further analysis of the chemical composition of the gaseous fraction was not carried out; however, a previous study found that most of the gases obtained during lignin microwave-assisted pyrolysis at lower temperatures were CO_2_, CO, CH_4_, and H_2_, with CO_2_ being, by far, the main product [16].

**Figure 6:**
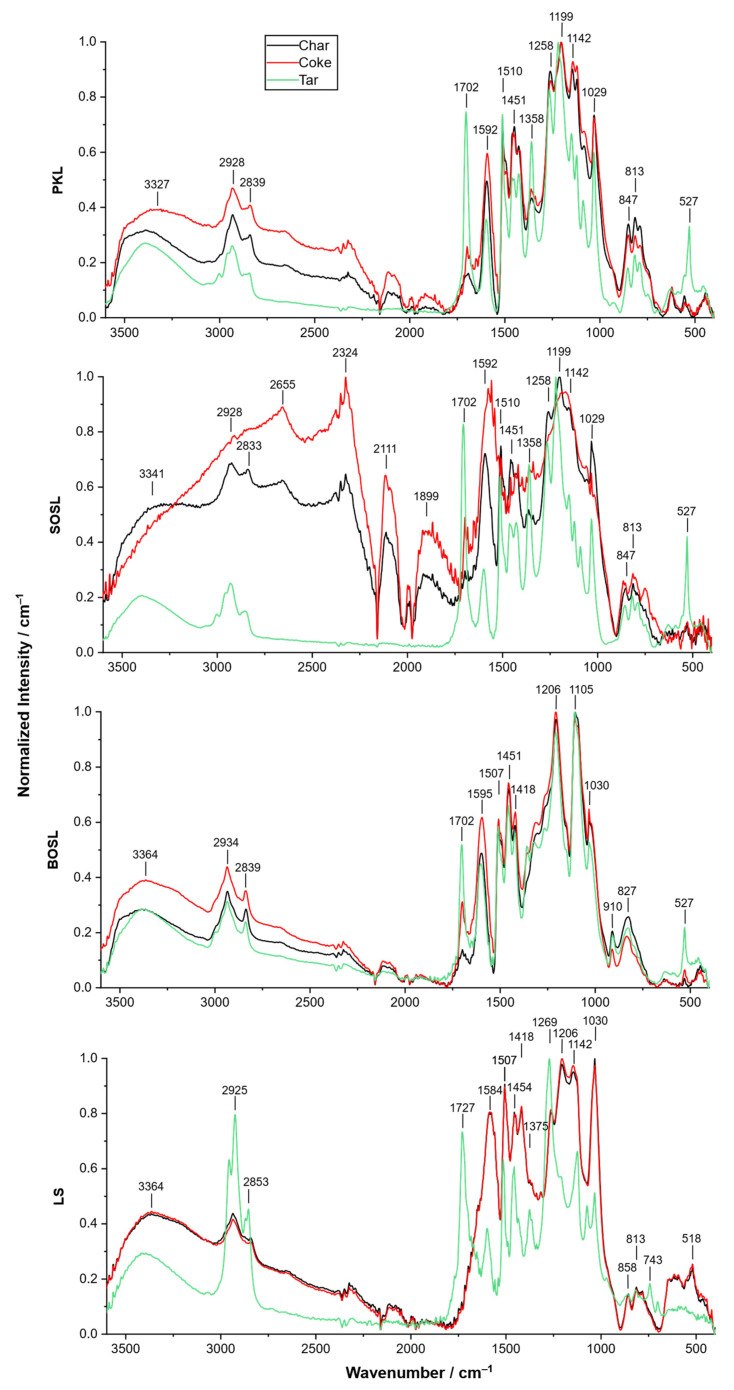
Normalized FTIR spectra of the chars (black), cokes (red), and tars (green) of the PKL, SOSL, BOSL, and LS.

As mentioned above, pyrolysis of the BOSL produced significantly larger amounts of tar compared to the other lignin types. To explain these results, the chemical nature of the chars and cokes have to be discussed in further detail. For this, the FTIR spectra of the char, coke, and tar of the different lignins were recorded (Figure 6). The considerations regarding the results obtained with FTIR concerning the signal overlap due to the high number of different functional groups described earlier was also valid for these results.

The spectrum of the SOSL coke differed from the spectra of the coke of the other lignins in a way that different signals were pronounced. Especially, those between 2650 and 1590 cm^−1^ had a high normalized intensity.

In all spectra of the cokes and chars, signals occurred at similar wavenumbers, indicating that the chemical structures of the different chars and cokes resembled each other. However, the intensity of the signals varied, outlining that the functional groups were differently abundant due to the differences in their chemical structures. Only for the LS, were the spectra of the char and the coke almost identical. For all the lignins, the normalized signals at higher wavenumbers of 3600–1800 cm^−1^ were more pronounced for the cokes than for the chars. Similarly, the intensities at 1580–1600 cm^−1^ were comparably higher for the cokes.

Meanwhile, comparing the spectra of the chars and cokes to those of the tars indicated more differences between the structures. For the PKL, SOSL, and BOSL, the normalized signals between 3600 and 1800 cm^−1^ were more pronounced for the cokes and chars than for the tars. This was not the case for the tar of LS, where strong signals occurred at 2925 and 2853 cm^−1^. However, the other signals at higher wavelengths were also less pronounced for the tar of LS compared to the coke and char. All of the tars had a pronounced signal at approximately 1702 cm^−1^, possibly originating from C=O groups. The tars of the PKL, SOSL, and BOSL produced an additional strong signal at approximately 527 cm^−1^, which could not be assigned.

Proposed assignments of the signals are listed in Table 2.

The char and coke of LS had almost identical spectra. This can be explained by the observation that only small amounts of tar could be extracted from the char. Thus, the extraction did not change the chemical composition of the char significantly, and the materials were almost identical. The small amount of tar that was extracted, however, had a highly different structure than the tar and coke. It contained a higher proportional content of CH_3_ or CH_2_ (2940–1830 cm^−1^) as well as C=O groups (1727 cm^−1^). In general, the ratio of aromatic and functional groups was higher than OH groups, which were cleaved by the pyrolysis as indicated by the lower signal at 3364 cm^−1^.

For the PKL and BOSL, the normalized spectra of the cokes had a higher intensity at higher wavenumbers than those of the chars, indicating that the ratio of functional groups in the cokes after acetone extraction changed. The signals at lower wavenumbers mainly originated from the aromatic structures in lignin, such as guaiacol structures, and were more pronounced for the chars. The signals at higher wavenumbers, which originated from hydroxy groups or water and unspecific methyl or methylene rests had a higher normalized intensity in the cokes than in the chars. Thus, mainly aromatic groups were extracted from the char, leading to a lower relative intensity in the coke. This is in line with the spectra obtained from the tars, which had a higher relative intensity at lower wavelengths, an indication of a high number of aromatic groups in the tars. The high viscosity of the tars provides additional information on the extracted compounds. The monomeric aromatic compounds won as bio-oil are often liquids with lower viscosity. Thus, the extracted tar contained aromatic structures that were not cleaved completely to the monomers but remained as oligomers or small polymers, explaining the higher viscosity. These molecules had boiling points that were too high for evaporation during the pyrolysis process but they could be extracted by acetone.

The spectra of the char and coke of the SOSL were different compared to each other, as high-intensity signals for the coke were found at 2650, 2321, 2115, and 1909 cm^−1^. The signals at wavenumbers of 1650 and 1909 cm^−1^ could not be assigned; in the literature on FTIR of lignin, they were not described as typically occurring signals. The overall signal intensity of the coke of the SOSL was lower than for the other measurements, indicating a large decrease in functional groups measured by FTIR. This can be derived from the highly normalized intensity of atmospheric carbon monoxide and carbon dioxide (2115 and 2321 cm^−1^, respectively) compared to the signals of the material itself. Hence, artifacts and false positives could be a larger factor, and further conclusions from the FTIR of the tar cannot be drawn. As for the other lignins, the spectrum of the tar indicates a high amount of functional and aromatic groups in the extracted product.

As mentioned above, the tar yield of the BOSL was much higher than that of the other lignins. The reason for this could be that unlike the SOSL and PKL, no porous larger network was formed during the pyrolysis of the BOSL. Thus, the final pyrolysis products had lower molecular sizes and were more soluble in acetone. The reason for the lower number of newly formed bonds could be the lower reactivity of the S-type lignin, which is more common in hardwood.

The low yield of phenolic condensates (6.0%) leads to the conclusion that a large part of the remaining oligomers could not be further cleaved under the given parameters.

**Figure 7:**
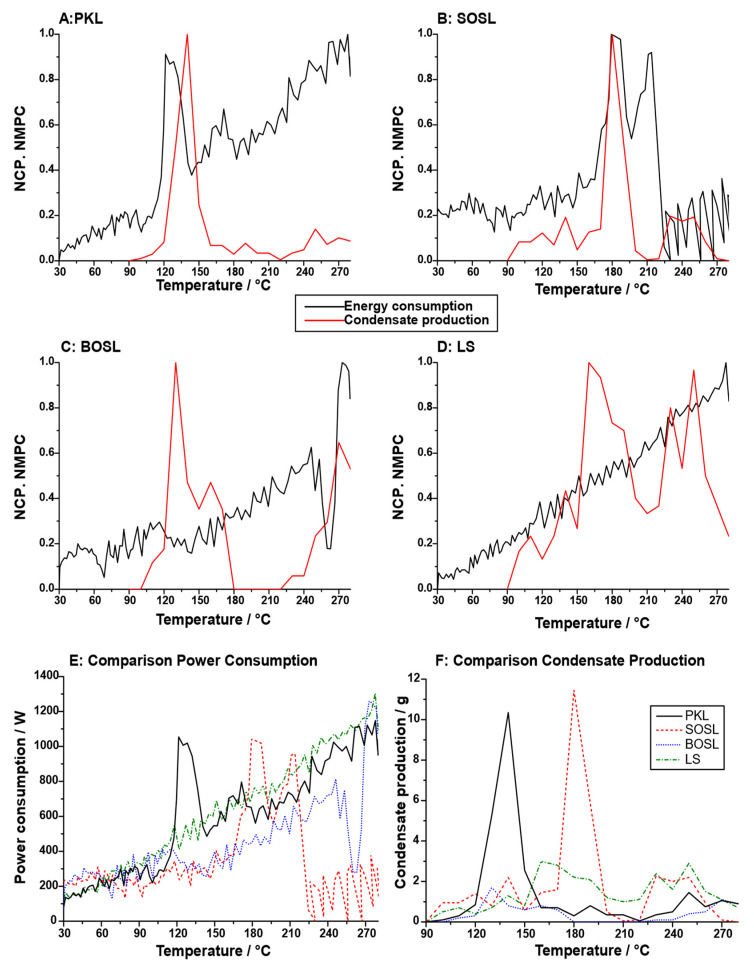
Normalized condensate production (NCP; red) and normalized microwave power consumption (NMPC; black) of the (**A**) PKL, (**B**) SOSL, (**C**) BOSL, and (**D**) LS as well as (**E**) a comparison of the microwave power consumption/W and of (**F**) the condensate production/g for the PKL (black; full line), SOSL (red; dashes), BOSL (blue; dots), and LS (green; dashes–dots).

For potential applications and further insight into the pyrolysis process, not only the overall mass balances but also the temperature at which the condensates were obtained and the energy needed to pyrolyze the lignin should be considered. To obtain an estimate of this, the normalized condensate production rate and the microwave power consumption were determined (Figure 7A–D). The normalized condensate production rate varied significantly between the different technical lignins. While for both the PKL and SOSL, a clear maximum could be determined (PKL: 140 °C; SOSL: 180 °C), both the BOSL and LS condensate production was high at two different temperatures (BOSL: 130 °C and 270 °C; LS: 160 °C and 250 °C). The overall microwave energy consumption was lower for the OSLs than for the LS and PKL (Figure 7E). The reason for this is that at high temperatures, for both the OSLs, the microwave power consumption decreases significantly, as exothermic reactions take place.

The high phenolics yields make SOSL and the PKL the most interesting lignins for applications in which phenolics are the desired products, which is the aim of most lignin cleavage processes. Of these two, the PKL could be considered more suitable, because the condensates can be obtained at a lower temperature, reducing the energy and time needed for production. However, the exothermic reaction that occurs at higher temperatures during the cleavage of both the SOSL and BOSL could reduce the energy needed for cleaving these lignin types. This is especially the case for the SOSL. Using this exothermic reaction could be a way to decrease the energy consumption of the process; however, potential corrosion of equipment due to the high temperature and high pressure has to be considered. The average energy consumption during the pyrolysis process of the PKL was calculated to be 0.53 kWh. Due to the exothermic reactions, those of the SOSL and BOSL were 0.31 and 0.37 kWh, respectively. This underlines the potential of using the exothermic reaction step to produce bio-oil at lower energy consumption. The energy consumption of the LS was the highest, on average at 0.56 kWh despite the overall low cleavage reactions.

As mentioned above, both the PKL and SOSL had a temperature at which a high fraction of the condensates was produced (Figure 7F). Thus, the bonds that were cleaved for condensate release were most probably of a similar nature. Microwave-assisted pyrolysis of lignin occurs in three degradation stages: first, the initial stage, during which the moisture is released from the lignin; afterwards, the second stage of fast degradation occurs, during which the *β*-O-4 bonds as well as other ether bonds are cleave, while side chains are also released and small oxygenated molecules, such as furans, acids, and ketones are formed; during the third stage, carbon–carbon bond breaking is the dominant reaction [16]. Considering the low temperatures of the cleavage, the main signal herein was expected to originate primarily from the second stage.

While for both the SOSL and the PKL a clear maximum condensate production at a given temperature was observed, the BOSL and the LS had several local maxima. During the pyrolysis of the BOSL, almost no aqueous phase was produced. Hence, the two local maxima of condensate production most probably both originated from phenolics production. This indicates that unlike the PKL and the SOSL, there were either two separate cleavage steps of one starting structure or that there were two different chemical compounds in the starting material. However, the overall condensate production was very low, and the normalization led to the over-exaggeration of the peaks as is visible in Figure 7F. Despite the very low condensate production, there was a decrease in the microwave power consumption at higher temperatures of the BOSL pyrolysis, indicating an exothermic process.

The condensate production rate of the LS has to be considered separately from the other lignin types. Since almost no phenolic fraction was produced, the LS was the only lignin with aqueous products as the main cleavage products. Hence, different cleavage reactions have to also be expected. The condensate production rate indicated that the cleavage products were produced without a clear maximum but at several temperatures with similar rates. Thus, it is likely that the condensates originated from various cleavage reactions, for example, cleavage of sulfonate groups and hydroxy groups.

It has to be mentioned that the temperature was measured with an external IR thermoelement. Thus, the temperature was measured on the surface of the sample, resulting in potential inaccuracies. Microwave heating results in a higher temperature in the middle of a sample [10]. Thus, the true temperature in the sample is expected to be higher than measured and described. In addition, due to the differences in microwave acceptance and sample position, local hot spots can occur. This effect can be further strengthened by heterogeneity due to the formation of char [31]. This may lead to significantly higher temperatures at hot spots than in the overall material, increasing cleavages at these spots.

**Figure 8:**
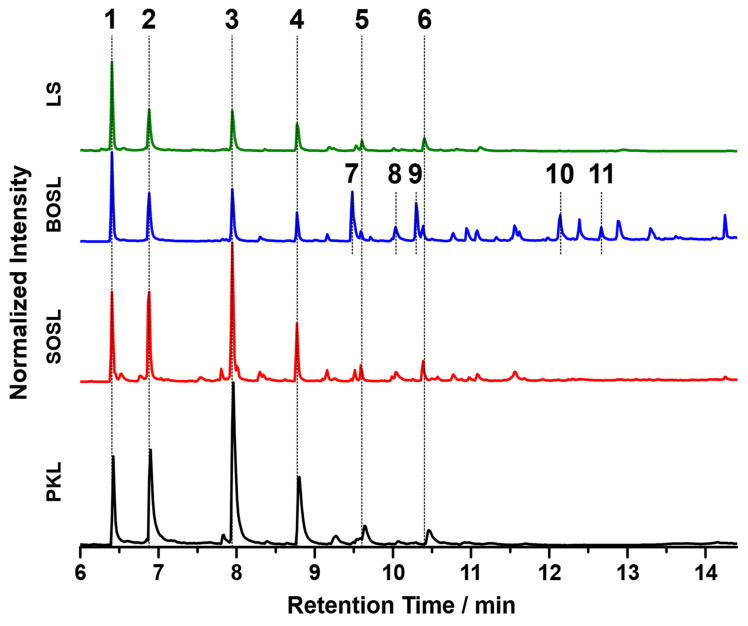
GC-MS chromatograms of the bio-oils won from the four technical lignins: PKL, SOSL, BOSL, and LS. The numbers in the figure refer to the proposed assignments in Table 3.

For comparison of the organic cleavage products, the chromatograms of the phenolic fractions of the four lignins are depicted in Figure 8. The tentative assignments of signals by GC-MS are listed in Table 3. For a better comparison, the chromatograms were normalized to the signal of the internal standard iodobenzene (signal 1 in Figure 8). The three bio-oils from the PKL, SOSL, and LS, all originating from softwood lignins, had very similar products. While the bio-oil of the BOSL originating from hardwood lignin also contained these products, and its chromatogram had significantly more different signals with similar high intensities. The five main products of the softwood lignins according to the proposed assignments were 2-methoxyphenol (signal 2; guaiacol), 4-methylguaiacol (signal 3), 4-ethylguaiacol (signal 4), 4-propylguaiacol (signal 5), and 4-prop-1-enylguaiacol (signal 6). The signal intensity varied significantly depending on the lignin source. As such, the signals of the PKL and SOSL, compared to the internal standard, had a much higher intensity than those of the LS. The results indicate that the pyrolysis of softwood lignins produces clear main products, while the other side products were only obtained in small quantities. The pyrolysis of hardwood lignin yielded a more distributed bio-oil composition of various compounds. Some of the additional compounds detected for the bio-oil of the BOSL were proposed as 2,6-dimethoxy-phenol (signal 7), syringaldehyde (signal 10; 4-hydroxy-3,5-dimethoxy-benzaldehyde), and acetosyringone (signal 11; 4′-hydroxy-3′,5′-dimethoxy-acetophenone). Additionally, for both the organosolv lignins, vanillin (signal 8; 4-hydroxy-3-methoxybenzaldehyde), one of the few products that was commercially won from lignin, was detected [32].

The proposed assignments of the signals visible in the chromatograms in Figure 8 are listed in Table 3. The signals having a high intensity only in the chromatogram of the bio-oil of the BOSL are marked with a (B) in the “Number in Figure 8” column.

The main reason for the more diverse composition of the bio-oil of the BOSL was the composition of the hardwood lignins. While softwood lignin is often mainly built from G-type precursors, the additional S-type precursors in hardwood upon cleavage produce compounds such as syringaldehyde or 2,6-dimethoxyphenol. These chemicals have both ortho-positions occupied with methoxy groups, as is also the case in the precursor of S-type lignin: sinapyl alcohol [3].

The chromatograms of the three softwood lignins indicated that the method applied for cleaving the lignins had a high selectivity towards certain bonds in the lignin structure, leading to the production of a few compounds with a high yield. This could make the used method interesting for applications aimed at obtaining these specific cleavage products.

**Figure 9:**
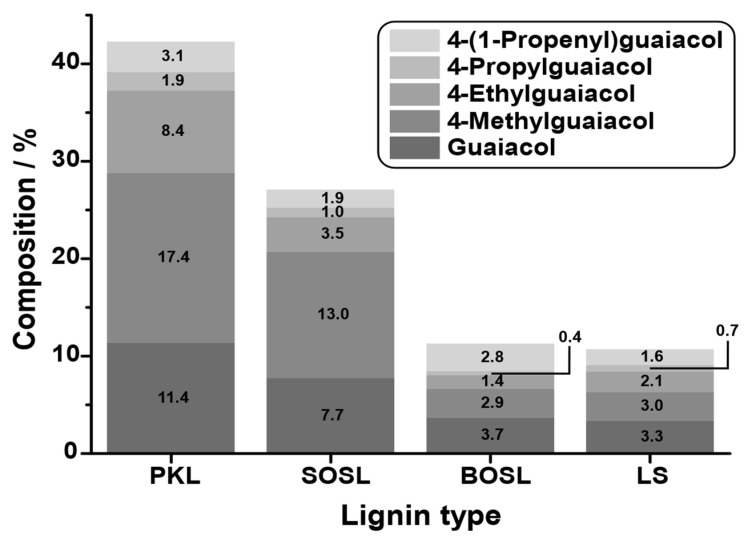
Mass percent of the main chemical products guaiacol (very dark grey), 4-methylguaiacol (dark grey), 4-ethylguaiacol (grey), 4-propylguaiacol (light grey), and 4-(1-propenyl)guaiacol (very light grey) in the phenolic pyrolysis fraction of the four technical lignins: PKL, SOSL, BOSL, and LS.

**Figure A1:**
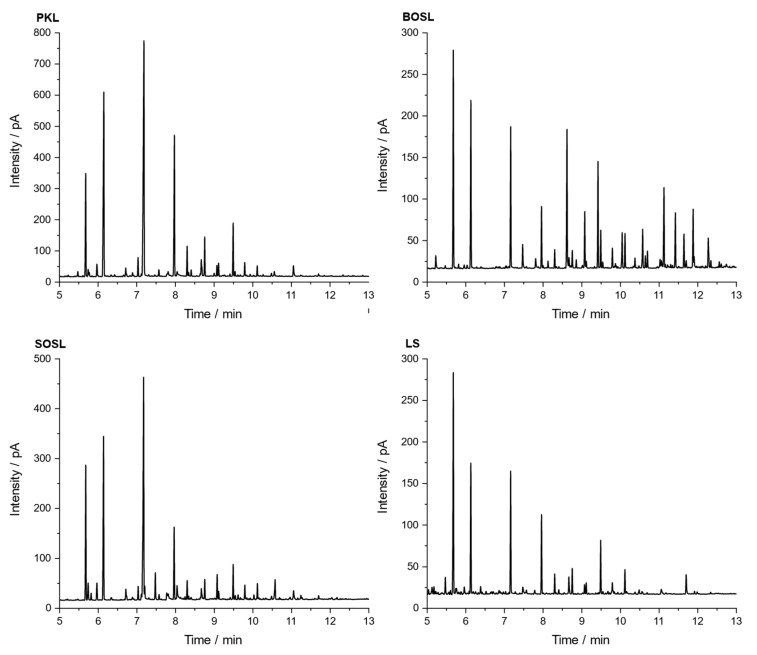
Chromatograms of the four bio-oils obtained by GC-FID.

To compare the quantities of small aromatic compounds in the bio-oil, the five main products that could be detected in all lignins, namely, guaiacol, 4-methylguaiacol, 4-ethylguaiacol, 4-propylguaiacol, and 4-(1-propenyl)guaiacol, were selected, and their quantity was determined by GC-FID. The resolution and peak width of the signals obtained by GC-FID were less broad and more symmetrical than those obtained by GC-MS (compare Figure A1 in Appendix A). This minimizes the effects of the overlap, leading to a more precise quantification. The results are depicted in Figure 9. The quantity of the main products varied significantly depending on the lignin source. While for the PKL, the five selected main products accounted for approximately 42% of the phenolic fraction composition, this value decreased for the SOSL, BOSL, and LS in that order, namely, being approximately 28%, 12%, and 11%, respectively.

The results indicate that a higher phenolic fraction yield also results in a higher percentage of monomers in the bio-oil. Thus, it is likely that the main difference in the yields originated from more monomers being cleaved overall. Due to the low yield of the selected monomers, especially in the BOSL and LS bio-oils, many additional compounds have to be part of the bio-oil’s composition. In the case of the BOSL, some of these compounds were already specified in the GC-MS; however, the low yield of the quantified molecules indicates that even more side products must be present in all bio-oils. These undetectable compounds could have a very low boiling point or a boiling point higher than the temperature to which the GC was heated (250 °C). However, after the liquid–liquid separation, the bio-oil was purified from the separation agent by evaporation. At this point, other also easily evaporated compounds were separated from the bio-oil. Thus, compounds with a low boiling point can be excluded, and the remaining compounds in the bio-oil must be compounds with a high boiling point. These compounds could be lignin oligomers, which are carried into the condensation system by other evaporating compounds, by the vacuum, or by the increasing pressure in the reactor due to the gas formation. The loss of oligomers in the reactor due to the fact of any of these reasons could additionally decrease the monomeric cleavage product yield of the reaction, as they are not cleaved further once carried into the condensation system.

Considering the selection of the cleavage products for quantification, the obtained yield of monomeric cleavage products for the BOSL was underestimated with this method. The true monomeric yield for the BOSL is expected to be closer to that of the SOSL due to the additional S-type lignin originating monomers.

### 3.5. Temperature-Dependent Condensate Analysis

**Figure 10:**
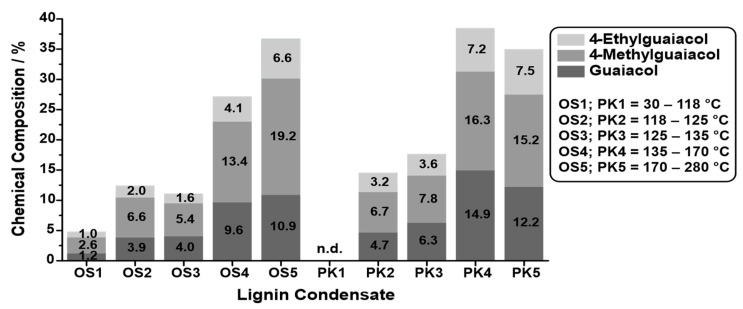
Mass percent of the main products guaiacol (dark grey), 4-methylguaiacol (grey), and 4-ethylguaiacol (light grey) in the condensates of the microwave-assisted pyrolysis of the SOSL (OS) and PKL (PK) at 30–118 °C (OS1/PK1), 118–125 °C (OS2/PK2), 125–135 °C (OS3/PK3), 135–170 °C (OS4/PK4), and 170–280 °C (OS5/PK5).

**Figure A2:**
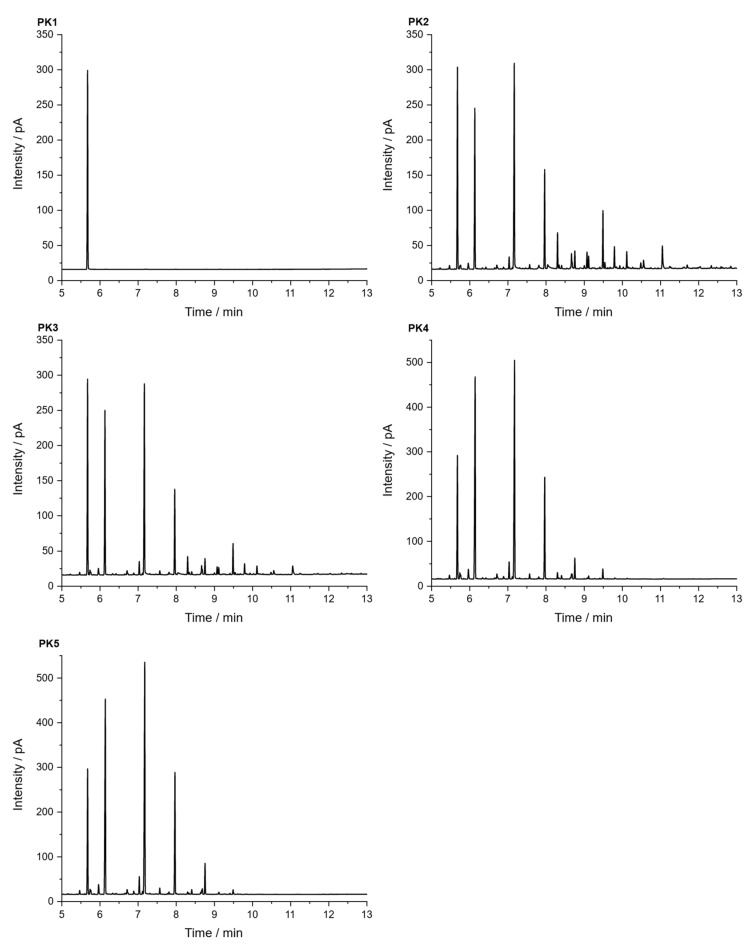
GC-FID chromatograms of the temperature series condensates of the pine kraft lignin.

**Figure A3:**
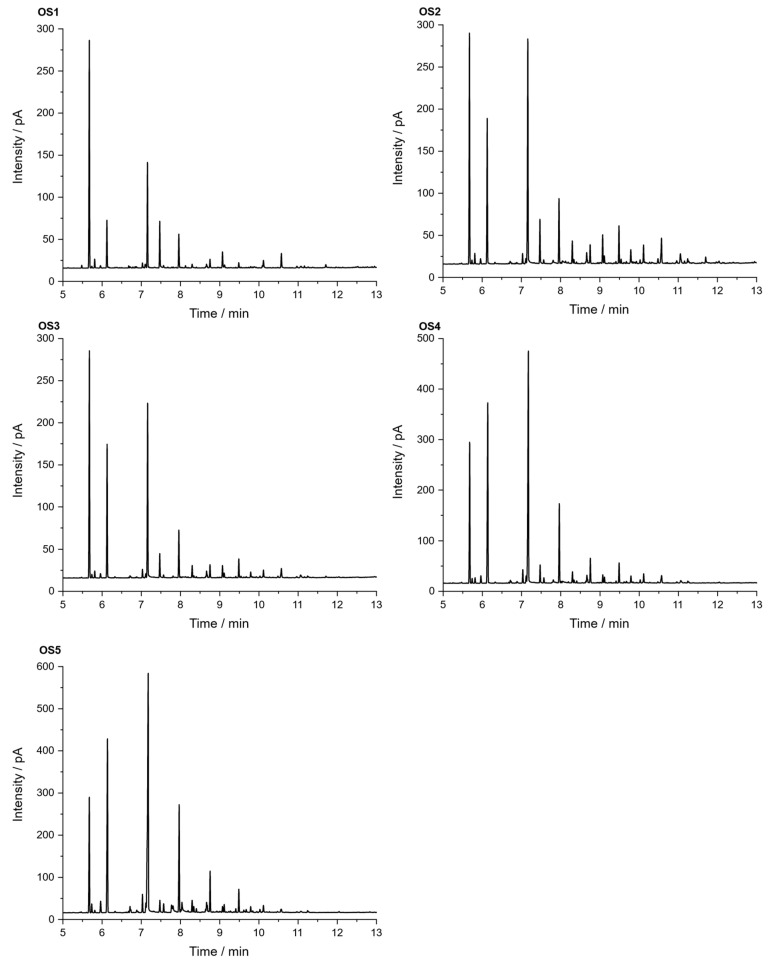
GC-FID chromatograms of the temperature series condensates of the spruce organosolv lignin.

For further insight into the pyrolysis process, condensate samples were collected at different temperatures during the pyrolysis process (Figure 10). The GC-FID chromatograms of the temperature-dependent condensates are depicted in Figure A2 and Figure A3 in Appendix A. The lignins selected for these experiments were the SOSL and PKL due to the fact of their high phenolic fraction yield. The amounts of 4-ethylguaiacol, 4-methylguaiacol, and guaiacol, the three main components of the phenolics, in the condensates were compared. For the SOSL, the general trend was that at higher temperatures, the condensate contained a higher amount of the selected monomers. The only exception was a higher monomer yield at 118–125 °C compared to the yield at 125–135 °C.

During the pyrolysis of the PKL not enough organic condensates for quantitative evaluation were collected during temperatures of 30–118 °C. As observed for the SOSL, higher temperatures led to condensates containing more monomers. However, the highest monomer yields were observed at a temperature range of 135–170 °C, slightly higher than for 170–280 °C.

The highest condensate production for the SOSL occurred at 180 °C. At this temperature, the highest monomer yields were also obtained, indicating that the high condensate production mainly stemmed from the phenolic monomers. These results indicate that the aqueous fraction was mainly produced at lower temperatures than the organic cleavage products.

The results for the PKL are in line with those of the SOSL: aqueous products are cleaved mainly at lower temperatures, while higher temperatures lead to more organic products. A major difference is, however, that the highest condensate production of the PKL occurs at lower temperatures than that of the SOSL, namely, at 140 °C. This phase of highest condensate production, as described for the SOSL, was mainly caused by the cleavage of organic compounds as indicated by the high organic monomer production.

The result that a higher temperature results in more organic and less aqueous yields is in line with a study by Farag et al. (2016) [10], who found that less energy is required to cleave hydrogen groups than organic monomers [10].

### 3.6. Comparison of the Results to Previous Studies

With vacuum low-temperature, microwave-assisted pyrolysis up to 280 °C, bio-oil yields of approximately 15–16% were obtained for the PKL and SOSL. Comparing these results to the literature was a difficult task, because a uniform definition of a bio-oil is lacking. While in this study, the bio-oil was separated from the aqueous phase, in some publications, “bio-oil” is defined as all condensates obtained from pyrolysis [14,16,18].

Bio-oil yields reported in the literature do not exclusively depend on pyrolysis temperature, as both lower and higher yields are reported over a broad range of temperatures [10,15,18,19]. However, the results in this work were compared to those in the literature of microwave-assisted pyrolysis at temperatures below 500 °C. Bu et al. (2014) [17] as well as Bartoli et al. (2020) [18] reported bio-oil yields above 40% at a 450 °C pyrolysis temperature, conducting the experiments under N_2_ gas flow or vacuum, respectively. It has to be considered that in their case, the bio-oil was not separated from the aqueous phase, resulting in a high water content of approximately 20–30%. However, even after separation of the aqueous products, these yields would be significantly higher than those reported in this paper. The reason for this could be the low temperature of the pyrolysis applied in this work, which could also be an advantage in terms of economic feasibility. Bu et al. reported the main bio-oil products to be guaiacols and other phenols, while Bartoli et al. obtained a high yield of aliphatic compounds and an aromatic content of approximately 53%, mainly guaiacols [17,18]. Considering that in this study, guaiacols were the main aromatic products, these results are comparable to those presented by Bu et al. [17]. However, it has to be stated that the aromatic content reported by Bartoli et al. was significantly lower than that obtained herein. Instead, many aliphatic cleavage products were obtained [18]. This underlines the importance of choosing a suitable pyrolysis method depending on the desired product composition. Nde et al. (2021) [19] reported bio-oil yields of a maximum 11% at 500 °C, which is a value more similar to those in this publication. The pyrolysis was conducted under vacuum. The bio-oil was defined as the condensates after pyrolysis without further separation. The main organic products described were guaiacols and phenols, which is in agreement with the results herein [19].

Summarizing, it has to be said that the bio-oil yield in this publication was significantly lower than some previously reported values. However, the low process temperature and the high specific monomer yield can be an advantage over other methods. Additionally, unlike in many other publications, no catalyst or microwave-absorbing material were used, which could further simplify the process. Economic considerations, as well as the desired products, could be crucial factors for the selection of one of the processes.

## 4. Conclusions

Vacuum low-temperature, microwave-assisted pyrolysis is an approach to obtaining phenolic cleavage products from technical lignins at lower temperatures than commonly described in the literature. Pyrolysis was carried out on four technical lignins. With the described method, a phenolic fraction was obtained from all four technical lignins. However, the yields of the phenolic fraction were highly dependent on the origin of the lignin. As such, the phenolic fraction yield of the PKL was almost ten times higher than that of LS. The PKL and SOSL were the lignins with the highest phenolic fraction yields, followed by the BOSL and LS. The PKL and SOSL had a clear maximum of condensate productions at 140 and 180 °C, respectively. The main products of all softwood technical lignins described herein were similar, namely, guaiacol and guaiacol derivates. The BOSL had a broader range of main products, partly originating from the S-type lignin structures typical of softwood. The concentration of the monomeric content in the phenolic fraction correlated positively with the bio-oil yield.

Temperature-dependent condensate analysis indicated that with increasing temperature, the obtained condensate contained more phenolic monomers. The ratio between the obtained phenolic monomers did not change significantly.

The low cleavage temperature of the lignin achieved herein indicates the potential of the described method; however, due to the low bio-oil yield, further research could be beneficial.

## Figures and Tables

**Table 1:** Signal maximum wavenumber, proposed assignment of the signal, and literature source for the assignment of the four technical lignins (see Figure 2).

Signal Wavenumber/cm^−1^	Proposed Assignment	Reference
3388	O-H stretching	[20,21]
2940–2830	C-H stretching in R-CH_3_ and R-CH_2_	[20,21]
1706	Unconjugated C=O stretching	[20,21]
1599	Aromatic skeletal vibrations (S- > G-type lignin) and C=O stretching	[20,21]
1511	Aromatic skeletal vibrations (G- > S-type lignin)	[20,21]
1457	C-H deformations in R-CH_3_ and R-CH_2_ (lignin or polysaccharides)	[20]
1425	CH3 bending vibration in lignin	[22]
1367	Aliphatic C-H stretching in CH_3_; phenolic OH	[21]
1319	S-ring plus G-ring condensed	[21]
1266	Guaiacyl ring breathing with carbonyl stretching	[23]
1210	C-C and C-O stretching in G-type lignin	[20]
1141	C-H in the plane deformation of guaiacyl ring and/or phenolic O-H	[23,24]
1110	Aromatic C-H in-plane deformation (S-type lignin)	[20]
1033	C=O stretching in lignin and/or aromatic in-plane deformation (G-type lignin)	[20,23]
965	HC=CH out of plane deformation	[21]
911	C-H out of plane in hardwood lignin	[25]
855	C-H out of plane, G-type units	[21]
812	C-H out of plane	[23]
649	S-O units	[26]
517	Unassigned	

**Table 2:** Signal maximum wavenumber, proposed assignment of the signal, and literature source for the assignment of the coke and char of the four technical lignins (see in Figure 6).

Signal Wavenumber/cm^−1^	Proposed Assignment	Reference
3350	O-H stretching	[20,21]
2930–2830	C-H stretching in R-CH_3_ and R-CH_2_	[20,21]
2655	Unassigned	
2324	Free carbon dioxide	[29]
2111	Free carbon monoxide	[30]
1899	Unassigned	
1702	Unconjugated C=O stretching	[20,21]
1592	Aromatic skeletal vibrations (S- > G-type lignin) and C=O stretching	[20,21]
1510	Aromatic skeletal vibrations (G- > S-type lignin)	[20,21]
1451	C-H deformations in R-CH_3_ and R-CH_2_ (lignin or polysaccharides)	[20]
1418	CH_3_ bending vibration in lignin	[22]
1358	Aliphatic C-H stretching in CH_3_; phenolic OH	[21]
1258	Guaiacyl ring breathing with carbonyl stretching	[23]
1199	C-C and C-O stretching in G-type lignin	[20]
1142	Aromatic C-H in-plane deformation	[20]
1029	C=O stretching in lignin and/or aromatic in-plane deformation (G-type lignin)	[20,23]
847	C-H out-of-plane, G-type units	[23]
813	C-H out-of-plane	[23]
527	Unassigned	

**Table 3:** Signal assignments and retention times of the chromatograms depicted in Figure 8. (B) stands for signals mainly visible in the chromatogram of the bio-oil won from BOSL.

Retention Time/Min	Number in Figure 8	Molar Mass/g/Mol	Proposed Assignment
6.41	1	204	Iodobenzene (internal standard)
6.88	2	124	Guaiacol
7.94	3	138	4-Methylguaiacol
8.77	4	152	4-Ethylguaiacol
9.48	7 (B)	154	2,6-Dimethoxyphenol
9.59	5	166	4-Propylguaiacol
10.03	8 (B)	152	Vanillin (4-hydroxy-3-methoxybenzaldehyde)
10.30	9 (B)	168	Unassigned
10.39	6	164	4-Prop-1-enylguaiacol
10.94	(B)	182	Trimethoxytoluene
11.56	(B)	166	Acetovanillone (4′-hydroxy-3′-methoxyacetophenone)
12.15	10 (B)	182	Syringaldehyde (4-hydroxy-3,5-dimethoxybenzaldehyde)
12.39	(B)	194	4-Allyl-2,6-dimethoxyphenol
12.66	11 (B)	196	Acetosyringone (4′-hydroxy-3′,5′-dimethoxyacetophenone)

## Data Availability

The data presented in this study are available upon request from the corresponding author.

## References

[1] Bajwa D.S., Pourhashem G., Ullah A.H., Bajwa S.G. (2019). A Concise Review of Current Lignin Production, Applications, Products and Their Environmental Impact. Ind. Crops Prod..

[2] Wang Y.-Y., Meng X., Pu Y., Ragauskas A.J. (2020). Recent Advances in the Application of Functionalized Lignin in Value-Added Polymeric Materials. Polymers.

[3] Rinaldi R., Jastrzebski R., Clough M.T., Ralph J., Kennema M., Bruijnincx P.C.A., Weckhuysen B.M. (2016). Paving the Way for Lignin Valorisation: Recent Advances in Bioengineering, Biorefining and Catalysis. Angew. Chem. Int. Ed..

[4] Kai D., Tan M.J., Chee P.L., Chua Y.K., Yap Y.L., Loh X.J. (2016). Towards Lignin-Based Functional Materials in a Sustainable World. Green Chem..

[5] Vishtal A., Kraslawski A. (2011). Challenges in Industrial Applications of Technical Lignins. Bioresources.

[6] Liao J.J., Latif N.H.A., Trache D., Brosse N., Hussin M.H. (2020). Current Advancement on the Isolation, Characterization and Application of Lignin. Int. J. Biol. Macromol..

[7] De Wild P.J., Huijgen W.J.J., Gosselink R.J.A. (2014). Lignin Pyrolysis for Profitable Lignocellulosic Biorefineries. Biofuels Bioprod. Bioref..

[8] Wang Y., Wang Q., He J., Zhang Y. (2017). Highly Effective C–C Bond Cleavage of Lignin Model Compounds. Green Chem..

[9] Hu L., Pan H., Zhou Y., Zhang M. (2011). Methods to Improve Lignin’s Reactivity as a Phenol Substitute and as Replacement for Other Phenolic Compounds: A Brief Review. Bioresources.

[10] Farag S., Mudraboyina B.P., Jessop P.G., Chaouki J. (2016). Impact of the Heating Mechanism on the Yield and Composition of Bio-Oil from Pyrolysis of Kraft Lignin. Biomass Bioenergy.

[11] Bu Q., Chen K., Xie W., Liu Y., Cao M., Kong X., Chu Q., Mao H. (2019). Hydrocarbon Rich Bio-Oil Production, Thermal Behavior Analysis and Kinetic Study of Microwave-Assisted Co-Pyrolysis of Microwave-Torrefied Lignin with Low Density Polyethylene. Bioresour. Technol..

[12] Arapova O.V., Chistyakov A.V., Palankoev T.A., Bondarenko G.N., Tsodikov M.V. (2020). Microwave-Assisted Lignin Conversion to Liquid Products in the Presence of Iron and Nickel. Pet. Chem..

[13] Farag S., Kouisni L., Chaouki J. (2014). Lumped Approach in Kinetic Modeling of Microwave Pyrolysis of Kraft Lignin. Energy Fuels.

[14] Morgan H.M., Liang J., Chen K., Yan L., Wang K., Mao H., Bu Q. (2018). Bio-Oil Production via Catalytic Microwave Co-Pyrolysis of Lignin and Low Density Polyethylene Using Zinc Modified Lignin-Based Char as a Catalyst. J. Anal. Appl. Pyrolysis.

[15] Yerrayya A., Suriapparao D.V., Natarajan U., Vinu R. (2018). Selective Production of Phenols from Lignin via Microwave Pyrolysis Using Different Carbonaceous Susceptors. Bioresour. Technol..

[16] Fan L., Song H., Lu Q., Leng L., Li K., Liu Y., Wang Y., Chen P., Ruan R., Zhou W. (2019). Screening Microwave Susceptors for Microwave-Assisted Pyrolysis of Lignin: Comparison of Product Yield and Chemical Profile. J. Anal. Appl. Pyrolysis.

[17] Bu Q., Lei H., Wang L., Wei Y., Zhu L., Zhang X., Liu Y., Yadavalli G., Tang J. (2014). Bio-Based Phenols and Fuel Production from Catalytic Microwave Pyrolysis of Lignin by Activated Carbons. Bioresour. Technol..

[18] Bartoli M., Rosi L., Frediani P., Frediani M. (2020). Bio-Oils from Microwave Assisted Pyrolysis of Kraft Lignin Operating at Reduced Residual Pressure. Fuel.

[19] Nde D.B., Muley P.D., Sabliov C.M., Nokes S.E., Boldor D. (2021). Microwave Assisted Pyrolysis of Kraft Lignin in Single Mode High-Q Resonant Cavities: Degradation Kinetics, Product Chemical Composition, and Numerical Modeling. Energy Convers. Manag..

[20] Kalami S., Arefmanesh M., Master E., Nejad M. (2017). Replacing 100% of Phenol in Phenolic Adhesive Formulations with Lignin. J. Appl. Polym. Sci..

[21] Faix O. (1991). Classification of Lignins from Different Botanical Origins by FT-IR Spectroscopy. Holzforschung.

[22] Harrington K.J., Higgins H.G., Michell A.J. (1964). Infrared Spectra of *Eucalyptus Regnans* F. Muell. and *Pinus Radiata* D. Don. Holzforschung.

[23] Faix O., Beinhoff O. (1988). Ftir Spectra of Milled Wood Lignins and Lignin Polymer Models (DHP’s) with Enhanced Resolution Obtained by Deconvolution. J. Wood Chem. Technol..

[24] Faix O., Böttcher J.H. (1993). Determination of Phenolic Hydroxyl Group Contents in Milled Wood Lignins by FTIR Spectroscopy Applying Partial Least-Squares (PLS) and Principal Components Regression (PCR). Holzforschung.

[25] Pandey K.K. (1999). A Study of Chemical Structure of Soft and Hardwood and Wood Polymers by FTIR Spectroscopy. J. Appl. Polym. Sci..

[26] Lima R.B., Raza R., Qin H., Li J., Lindström M.E., Zhu B. (2013). Direct Lignin Fuel Cell for Power Generation. RSC Adv..

[27] Brebu M., Cazacu G., Chirila O. (2011). Pyrolysis of Lignin—A Potential Method for Obtaining Chemicals and/or Fuels. Cellulose Chem. Technol..

[28] Nitsos C., Stoklosa R., Karnaouri A., Vörös D., Lange H., Hodge D., Crestini C., Rova U., Christakopoulos P. (2016). Isolation and Characterization of Organosolv and Alkaline Lignins from Hardwood and Softwood Biomass. ACS Sustain. Chem. Eng..

[29] Hesse M., Meier H., Zeeh B., Bienz S., Bigler L., Fox T. (2012). Spektroskopische Methoden in der Organischen Chemie.

[30] Socrates G. (2010). Infrared and Raman Characteristic Group Frequencies: Tables and Charts.

[31] Gadkari S., Fidalgo B., Gu S. (2017). Numerical Investigation of Microwave-Assisted Pyrolysis of Lignin. Fuel Process. Technol..

[32] Evstigneyev E.I., Shevchenko S.M. (2020). Lignin Valorization and Cleavage of Arylether Bonds in Chemical Processing of Wood: A Mini-Review. Wood Sci. Technol..

